# Chemical, rheological, and sensorial properties of Baladi bread supplemented with buckwheat flour produced in Egypt

**DOI:** 10.1038/s41598-023-48686-1

**Published:** 2024-02-07

**Authors:** Ahmed M. S. Hussein, Hala A. Abd El-Aal, Nahla M. Morsy, Mohamed M. Hassona

**Affiliations:** 1 Food Technology Department, National Research Center, Dokki, Cairo Egypt; 2 Department of Sustainable Development of Environment and Its Projects Management, Environmental Studies & Research Institute (ESRI), University of Sadat City (USC), Six Zone, Sadat City, Menofiya, 22897 Egypt; 3 The Qur’anic Botanic Garden, Hamad Bin Khalifa University (HBKU), Doha, Qatar

**Keywords:** Biochemistry, Biological techniques, Chemical biology

## Abstract

This research aimed to enhance the nutritional and sensory qualities of Balady bread by adding locally Egyptian buckwheat flours, *Fagopyrum esculentum* (FE) and *Fagopyrum tataricum* (FT), to Hard Wheat Flour (HWF) 82% extraction at three levels (10%, 20%, and 30%). The chemical composition, rheological properties, color, sensory evaluation and stalling of the balady bread were determined. The chemical composition of raw materials revealed that FE was significantly (*P* ≤ 0.05) higher in protein and fat contents compared to HWF and FT. While FT was higher in fiber and ash contents. The findings show that a 30% replacement with FE or FT significantly enhances the bread's nutritional profile, notably increasing protein, fiber, ash, and moisture content. Rheological analysis revealed that FE and FT alter dough handling, with a notable improvement in dough stability and mixing tolerance at 30% FT. Sensory evaluation indicated acceptable qualities even at higher substitution levels, although 30% FE showed slight declines in certain attributes. Furthermore, bread supplemented with 30% FT demonstrated slower staling and potentially extended shelf life. These results highlight the potential of FE and FT as nutritional enhancers in bread formulations, with 30% FT emerging as the optimal replacement level for balancing nutritional benefits and sensory acceptance.

## Introduction

Baladi bread holds great importance in Egypt as a staple food. Efforts have been made to improve its quality and extend its shelf life, with research suggesting that the addition of hydrocolloids, such as pullulan, can enhance the dough properties and delay staling of baladi bread^1^. However, Consuming buckwheat has also been associated with the reduction of hyperlipidemia, blood pressure, and improved weight regulation. It has been found to have a lower postprandial blood glucose and insulin response, making it beneficial for individuals with diabetes. Buckwheat's high levels of rutin and quercetin contribute to its antioxidative activity^2^ (Kreft). On the other hand, buckwheat is a highly nutritious food that offers various health benefits. Its bioactive compounds, such as D-chiro-inositol, buckwheat proteins, and flavonoids, contribute to its positive effects on cholesterol levels, neuroprotection, anticancer and anti-inflammatory properties, hypertension, and diabetes. Rutin, in particular, shows potential therapeutic applications for Alzheimer's disease^2,3^. Conversely, Hard Wheat Flour (HWF) primarily contains starch (55–75%), proteins (6–12%), lipids (1–4%), soluble carbohydrates (1–2%), total dietary fibers (3–7%), and ash (1–2%)^4^. Fagopyrum esculentum (FE), or common buckwheat, is rich in alkaloids, amino acids, anthraquinones, carbohydrates, flavonoids, phlobatannins, tannins, high-quality proteins, essential amino and fatty acids, fiber, vitamins, and minerals like iron, zinc, and selenium^4,5^. Fagopyrum tataricum (FT), or Tartary buckwheat, shares a similar chemical composition with FE, including high levels of flavonoids with antioxidant activity and nutritive compounds^6,7^. On the contrary, the quality of bread, particularly when wheat flour is supplemented, is critically assessed using farinograph and extensograph parameters. The impact of transglutaminase on dough with barley or soy flour was examined using a farinograph^8^. The influence of soy flour and Cephalariasyriaca flour on dough extensibility was explored by Mashayekh et al.^9^ and Karaoğlu^10^, respectively. Recombinant lipoxygenase was found to improve both farinograph and extensograph measures^11^, while mealworm powder was observed to reduce dough elasticity^12^. The effects of barley and maize flour on extensibility were also investigated, showing varied impacts^13,14^. However, Pasting properties of Hard wheat flour in Baladi bread can be influenced by ingredient addition and substitution. Abdel‐Kader^15^ found that enriching Baladi bread with decorticated cracked broad beans flour increased water absorption, arrival time, and dough development time. The addition of soy flour impacted the sensory and rheological properties of wheat bread^9^. Choi et al.^16^ discovered that substituting normal and waxy-type whole wheat flour decreased pasting viscosity. While, the chemical composition of Baladi bread has been diversified to explore the influence of various ingredients. Hadidy and Rizk^17^ focused on enriching fatty acids with coriander seeds. The elemental composition and sensory attributes of bread supplemented with barley and corn flours were evaluated by Hussein et al.^18^. Mohamed et al.^19^ enhanced bread with folic acid and whole grains, improving nutritional content. The enrichment of red algae was studied by Yousef et al.^20^, while Hussein et al.^21^ assessed low-protein variants. Ingredients like seed coats, rice bran, and watermelon rinds were experimented with to assess changes in chemical and sensory properties, as well as potential health benefits^22–25^. Also, the color attributes of Baladi bread can be altered by ingredients and processing techniques. Barley and corn flour supplementation changed bread color^18^, while guar gum breads mimicked French bread color^26^. Whey protein was noted to darken the crust of breads^27^. The influence of coriander seeds, nano-powders, and red algae on color parameters was highlighted^17,20,28^. The impact of watermelon rinds and rice bran was assessed^23,25^, and wheat grain fertilization techniques were compared^29^. However, olive oil amounts did not significantly change ciabatta bread color^30^. Lastly, Various additives' impact on Baladi bread's sensory properties and freshness during storage has been extensively studied. The addition of grape seed, quinoa, and breadfruit flours showed improvements in nutritional and sensory aspects^31–33^. Barley and red algae were observed for nutritional gains but with some sensory changes^18,20^. Watermelon rind flour could replace up to 15% of wheat flour without affecting sensory attributes^25^ . The role of enzymes and storage temperature on bread freshness was emphasized by Ghoshal et al.^34^ and El-Sayed^35^ while higher protein content and dairy by-products were linked to extended freshness and shelf-life^29,36^.

## Materials and methods

### Materials

#### Buckwheat varieties

In a study from 2018 to 2020, Fagopyrum tataricum and Fagopyrum esculentum were grown in Bilbies city, Sharkia and Sadat City, Monofiya in Egypt to examine their growth, productivity, and quality under different timings. Grains were harvested, mixed, dried, and purified. The buckwheat seeds, bought from a commercial company based in California USA, were airfreighted to Egypt, inspected internationally and nationally, passed quarantine checks, and underwent germination tests before planting in the study areas^34,37,38^.

The Authors obtained the necessary approvals from the Ministry of Agriculture and Land Reclamation (MALR) on importation and cultivation (in both mentioned locations) of Buckwheat seeds for the abovementioned cultivars. However, imported varieties also have got phytosanitary certificate issued by USDA and all the seeds delivered directly from the producer through airfreight, as a comply with relevant institutional, national, and international guidelines and legislation.

### Wheat flour and other ingredients

Hard Wheat flour (HWF) with an 82% extraction rate was sourced from the South Cairo Mill Company located in Giza, Egypt. Sugar, salt (sodium chloride), and yeast (Saccharomyces cerevisiae) were procured from the local market in Giza, Egypt, for use in the study.

### Chemicals and solvents

In this research study, all the chemicals and solvents employed were of analytical reagent grade. Specifically, the solvents used, including trichloroacetic acid (TCA), thio barbituric acid (TBA), and DPPH (2,2-Diphenyl-1-picryl-hydrazyl), were procured from the Gomhoria Company for Chemical and Trading, based in Cairo, Egypt.

## Methods

### Technological treatment

#### Preparation of flour mixtures

Buckwheat seeds were cleaned and moistened to 15% moisture content. Then, they were ground using a Quadrumat Junior flour mill and sifted through a 40-mesh sieve. The resulting flour was packed into plastic sachets. We mixed this buckwheat flour with hard wheat flour (HWF) with different proportions: 100% HWF, 90% HWF and 10% BWF, 80% HWF and 20% BWF, and 70% HWF and 30% BWF.

### Rheological properties of dough

We assessed the doughs' rheological characteristics using farinograph, extensograph, and Rapid-viscoanalyzer (RVA) tests following the AACC^39^ guidelines.

### Preparation of Baladi bread

Flour blends (Table 1) were combined with yeast, salt, and water and mixed for 6 min. The dough fermented for 1 h at 30 °C and 85% humidity. Afterward, 125 g pieces were rested on a bran-sprinkled board for 45 min. Each piece was then flattened to 20 cm in diameter, proofed for 15 min between 30 and 35 °C, and baked at 400–500 °C for 1–2 min. Baked loaves cooled for 1 h before evaluation.

**Table 1:** Proximate Chemical Composition (%) of Raw Materials (on dry weight basis).

Samples	Chemical composition of flour samples (%)					
	Moisture	Ash	Fiber	Protein	Lipids	CHO
HWF	13.02 ± 0.22	1.47 ± 0.0	1.65 ± 0.01	11.75 ± 0.28	1.81 ± 0.08	83.32 ± 0.78
FE	9.17 ± 0.17	2.58 ± 0.07	12.51 ± 0.32	14.90 ± 0.27	2.18 ± 0.05	67.83 ± 0.56
FT	8.78 ± 0.29	2.85 ± 0.01	22.08 ± 0.37	11.81 l ± 0.25	1.57 ± 0.03	61.69 ± 0.85

Where HWF: Hard wheat flour; FE (*Fagopyrum esculentum*), FT (Fagopyrum tataricum) Cho: total carbohydrate.

### Color attributes

The color characteristics (L, a, and b values) of various types of Baladi bread were measured using a Tristimulus Color Analyzer, specifically the Hunter Lab Scan XE, located in Reston, Virginia. This analysis was performed using a standard white tile as the reference point.

### Sensory evaluation

Trained panelists, a group of 15 individuals, conducted sensory evaluations of the Baladi bread loaves following the methodology outlined by Hussein et al*.*^18^. Each sample was assessed based on the following criteria:

1. General appearance (worth 20 points)
2. Layers separation (worth 20 points)
3. Roundness (worth 15 points)
4. Crumb distribution (worth 15 points)
5. Crust color (worth 10 points)
6. Taste (worth 10 points)
7. Odor (worth 10 points)

Panelists assigned scores to each of these attributes to evaluate the sensory characteristics of the Baladi bread loaves.

### Freshness of bread

The freshness of Baladi bread loaves was assessed after packaging them in polyethylene bags and storing them at room temperature for 1, 3, and 5 days. The assessment was conducted using the Alkaline Water Retention Capacity test (AWRC), following the procedure initially developed by Yamazaki^40^ and subsequently modified by Kitterman and Rubenthaler^41^.

### Formula of Baladi bread

The formula for making Baladi Bread with different proportions of Buckwheat flour (Fagopyrum esculentum and Fagopyrum tataricum) along with the control sample. The ingredients used in the formula are as follows:

- Hard wheat flour (HWF): 100% in the control sample, and varying percentages (90%, 80%, and 70%) in the samples with buckwheat.
- Buckwheat flour (Fagopyrum esculentum and Fagopyrum tataricum): 0% in the control sample, and 10%, 20%, and 30% in the samples with buckwheat.
- Dry yeast: 1.5 g in all samples.
- Salt: 1.0 g in all samples.

In summary, the formula details the proportions of these ingredients used to prepare Baladi Bread with varying levels of buckwheat flour, allowing for different formulations and experimental samples to be created.

### Analytical methods

#### Chemical composition

Moisture, protein, fat, ash, and crude fiber contents were assessed using the methods specified in the AOAC^42^ (Association of Official Analytical Chemists) 2005 guidelines. To determine the carbohydrate content, the following formula was applied:

Carbohydrates (%) = 100—(% protein + % fat + % ash + % crude fiber).

This calculation method subtracts the percentages of protein, fat, ash, and crude fiber from 100% to estimate the carbohydrate content in the samples.

### Statistical analysis

The collected results were statistically analyzed using an analysis of variance (ANOVA) method, as described in McClave and Benson's^43^ reporting.

## Results and discussion

### Chemical composition of raw materials

Table 1 shows that the Hard Wheat Flour (HWF) was high in moisture (13.02%) and carbohydrates (83.02%), aligning with (Al-Snafi^4^). In contrast, Fagopyrum esculentum and Fagopyrum tataricum (FE) had lower moisture but higher protein, ash, and fiber contents. Specifically, Fagopyrum esculentum (FT) had more protein (14.90%)^5^ while Fagopyrum tataricum was rich in fiber (22.08%) and ash (2.85%)^6,7^. These findings suggest their potential in enriching bread with essential nutrients.

### Rheological parameters

#### Farinograph parameters

**Figure 1:**
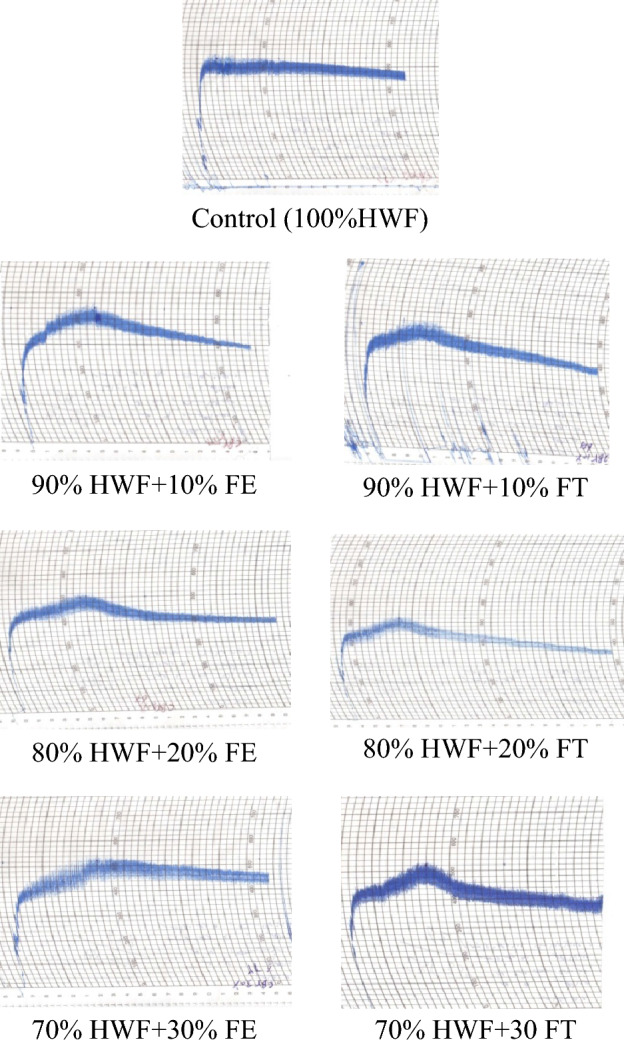
Farinograph parameters of dough sample supplemented with 10, 20, 30% *Fagopyrum esculentum* (FE) and *Fagopyrum tataricum* (FT) with Hard wheat flour (HWF).

Table 2 and Fig. 1 assessed the Farinograph parameters of HWF supplemented with FE. HWF's 100% control displayed specific values for water absorption, arrival time, and dough stability. Notably, 10% FT reduced water absorption and dough stability, increasing the mixing tolerance index significantly. At 30%, a more balanced dough behavior emerged. Conversely, FT supplementation led to decreased dough stability but higher mixing tolerance, particularly at 20%. These results reveal that alternative flour type and level can significantly impact Farinograph parameters, influencing dough handling and product quality^44^. The interplay between dough stability and mixing tolerance underscores the nuanced effects of supplementation on rheological properties, consistent with prior research^8^. Flour supplementation's profound impact necessitates precise understanding and balance for optimal bread formulations.

**Table 2:** Farinograph parameters of HWF supplementation with FE and FT.

Samples	Water absorption (%)	Arrival time (min)	Dough developme-nt time (min)	Dough stability (min)	Dough stability (min)	Weakening (BU)
Control (100% HWF)	66.4	1.0	2.5	2.5	20	20
90% HWF + 10% FE	65.00	1.5	6.0	6.0	80	110
80% HWF + 20% FE	64.00	2.0	6.0	6.0	60	110
70% HWF + 30% FE	63.00	2.5	7.0	7.0	20	50
90% HWF + 10% FT	64.00	2.0	4.5	4.5	60	100
80% HWF + 20% FT	63.00	2.5	5.0	5.0	80	130
70% HWF + 30% FT	62.00	2.5	5.0	5.0	90	100

Where: HWF: Hard wheat flour; FE: *Fagopyrum esculentum*; FT: *Fagopyrum tataricum*; BU: barabender unit.

### Extensograph parameters

**Figure 2:**
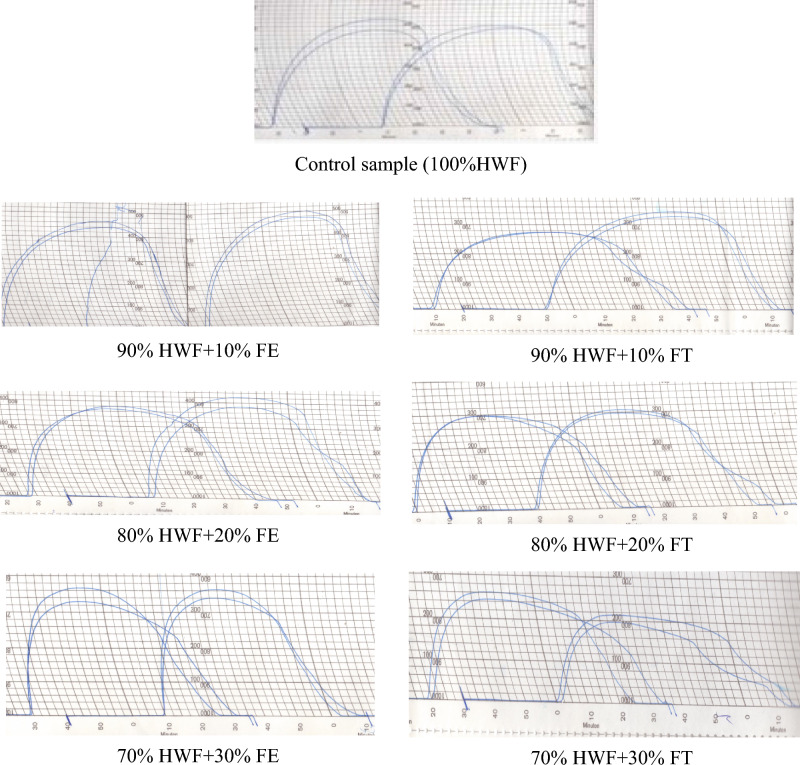
Extenograph parameters of dough sample supplemented with 10, 20, 30% *Fagopyrum esculentum* (FE) and *Fagopyrum tataricum* (FT) with Hard wheat flour (HWF).

Table 3 and Fig. 2 show that the extensograph profiles of dough made from Hard Wheat Flour (HWF) supplemented with Fagopyrum esculentum and Fagopyrum tataricum. At 10% Fagopyrum esculentum, extensibility and resistance improved, but at 30% levels, both metrics decreased dramatically. Meanwhile, 30% Fagopyrum tataricum resulted in a softer dough with reduced extensibility and resistance. These findings are consistent with previous studies that show the variable effects of different supplementations on extensograph parameters^9–14^. Our results indicate that the type and level of supplementation have a significant impact on dough's mechanical properties and ultimately on bread quality. Understanding these extensograph parameters in the context of alternative flour types is crucial for optimized bread formulations.

**Table 3:** Extensograph parameters of HWF supplementation with FE and FT.

Samples	Extensibility (E) (cm)	Resistance to extension (R) (BU)	Maximum resistance to extension (BU)	Proportional number (R/E)	Dough energy (cm2)
Control (100%HWF)	155	490	410	2.64	97
90% HWF + 10% FE	165	580	430	2.60	112
80% HWF + 20% FE	132	430	380	2.78	72
70% HWF + 30% FE	92	270	270	2.93	32
90% HWF + 10% FT	165	360	270	2.24	71
80% HWF + 20% FT	100	250	240	1.54	47
70% HWF + 30% FT	125	180	180	1.44	30

Where: HWF: Hard wheat flour; FE: *Fagopyrum esculentum*; FT: *Fagopyrum tataricum*; BU: barabender unit.

### Pasting profile (RVA)

**Figure 3:**
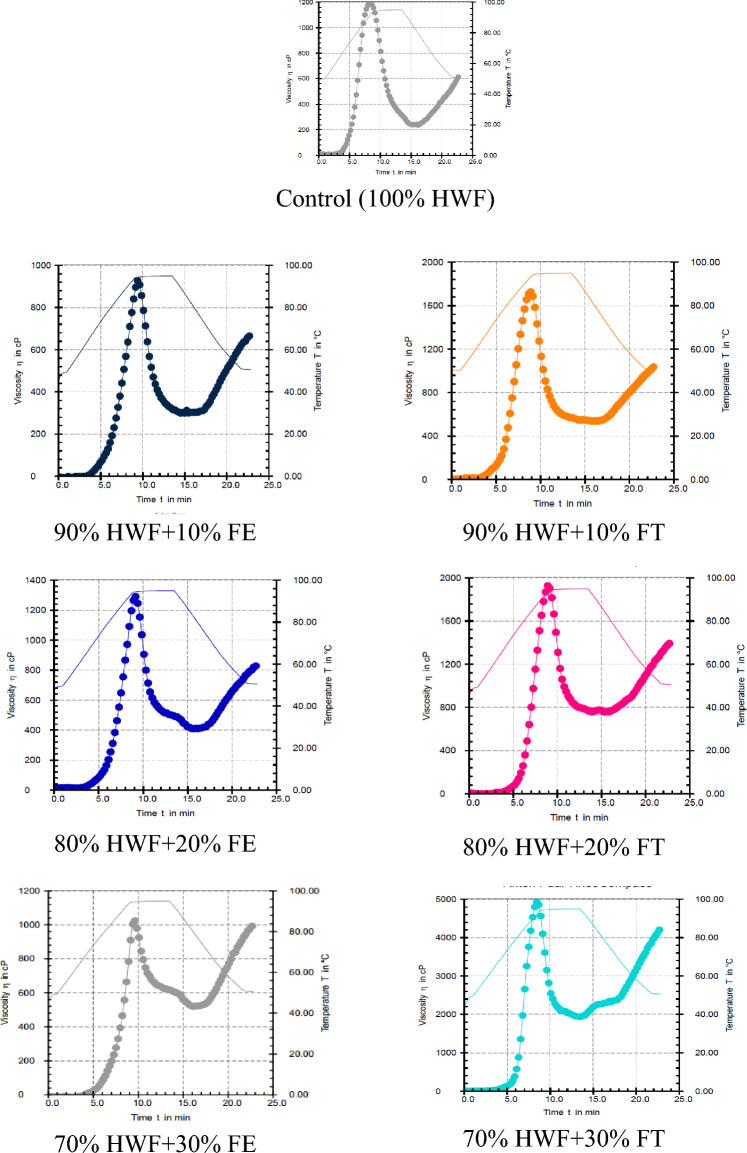
Pasting profile (RVA) of dough supplemented with 10, 20, 30% *Fagopyrum esculentum* (FE) and *Fagopyrum tataricum* (FT) with Hard wheat flour (HWF).

Our study examined how Fagopyrum esculentum and Fagopyrum tataricum supplementation affect the pasting properties of Hard Wheat Flour (HWF)-based baladi bread as Table 4 and Fig. 3. The control sample showed a peak viscosity of 1192 CP, which declined with Fagopyrum esculentum and rose significantly with Fagopyrum tataricum, peaking at 4949 CP at 30% supplementation. These findings echo earlier research on the influence of various ingredients like soy flour and decorticated cracked broadbeans on pasting properties^9,15^. Moreover, Choi et al.^16^ reported similar effects with whole wheat flour substitution. Pasting and peak temperatures also varied but stayed within narrow ranges. These alterations in pasting properties are crucial, as they provide insights into potential textural and sensory changes in the final bread product, affirming the significant role of ingredient supplementation in bread formulation.

**Table 4:** Pasting properties of HWF supplementation with FE and FT at different levels.

Samples	Peak Vis. (CP)	Trough1 (CP)	Break down (CP)	Final Vis. (CP)	Setback (CP)	Peak Time) Min)	Pasting Temp. (°C)	Peak Temp. (°C)
Control (100%HWF)	1192	887	956.7	630.7	561	8.23	56.7	91.8
90% HWF + 10% FE	927.1	297.05	630	671.5	255.6	9.37	57.3	94.4
80% HWF + 20%FE	1291	503.93	885.3	836.4	455	9.07	60.4	94.4
70% HWF + 30% FE	1028	518.31	509.3	999.2	489.8	9.53	68.1	94.5
90% HWF + 10% FT	1731	837.6	1196	1045	686.4	8.87	61.3	93.1
80% HWF + 20% FT	1928	755.4	1172	1406	521.3	8.9	58.5	93.6
70% HWF + 30% FT	4949	1939.8	3019	4242	706.8	8.5	63.5	92.5

Where: Where: HWF: Hard wheat flour; FE: *Fagopyrum esculentum*; FT: *Fagopyrum tataricum*; BU: barabender unit; Vis: viscosity; CP: centipoise.

### Chemical composition of Baladi bread

Table 5 shows that the chemical composition of Baladi bread supplemented with FE and FT showed significant enhancements in key nutritional metrics. Specifically, the bread with 30% FE or FT supplementation had notably higher levels of protein, moisture, ash, and fiber compared to the control made from 100% HWF in agreement with Hadidy and Rizk^17^ and Mohamed et al.^19^ that observed improvements in fatty acids and micronutrients like folic acid when using different additives. However, Hussein et al.^18^ extended this by examining mineral content, corroborating the increase in ash content seen in our study with FE and FT supplements. Also, Studies by Yousef et al.^20^ and Sandak et al.^22^ showed that unconventional additives like red algae and seed coats can also alter the bread's chemical composition.

**Table 5:** Chemical composition of Baladi bread supplemented with different levels of FE and FT (on dry weight basis).

Baladi bread	Moisture	Protein	Ash	Fat	Fiber	Carbohydrate
Control (100%HWF)	34.52e ± 1.65	11.60e ± 0.25	1.50d ± 0.06	1.62d ± 0.08	1.65 g ± 0.01	83.63^a^ ± 2.35
90% HWF + 10% FE	35.65^d^ ± 1.25	12.05^c^ ± 0.15	1.75^d^ ± 0.03	1.60^d^ ± 0.01	2.70f. ± 0.10	81.90^b^ ± 1.39
80% HWF + 20%FE	36.80^c^ ± 1.50	12.65^b^ ± 0.10	2.00^d^ ± 0.09	1.55^e^ ± 0.03	3.50^e^ ± 0.07	80.30^d^ ± 1.65
70% HWF + 30% FE	37.90^b^ ± 1.45	13.10^a^ ± 0.07	2.35^ab^ ± 0.11	1.52^e^ ± 0.05	4.06^c^ ± 0.13	78.97^e^ ± 1.35
90% HWF + 10% FT	36.00^d^ ± 1.60	11.65^e^ ± 0.11	1.80^ cd^ ± 0.02	1.70^c^ ± 0.07	3.95^d^ ± 0.12	80.90^c^ ± 1.15
80% HWF + 20% FT	37.50^b^ ± 1.70	11.70^d^ ± 0.09	2.10^bc^ ± 0.05	1.80^b^ ± 0.05	5.80^b^ ± 0.15	78.60f. ± 0.95
70% HWF + 30% FT	38.70^a^ ± 1.75	11.80^d^ ± 0.16	2.50^a^ ± 0.07	1.89^a^ ± 0.02	7.50^a^ ± 0.17	76.31^ g^ ± 0.89
LSD at 0.05	0.665	0.067	0.379	0.0579	0.0597	0.0585

Where: HWF: Hard wheat flour; FE: *Fagopyrum esculentum*; FT:* Fagopyrum tataricum.*

Results are presented as means for triplicate analyses ± standard deviation (SD). Means within column with different letters are significantly different (*P* ≤ 0.05).

### Color attributes of Baladi bread

The Table 6 explores the color attributes of Baladi bread supplemented with varying levels of Fagopyrum esculentum (FE) and Fagopyrum tataricum (FT). It highlights the impact of these ingredients on parameters such as L* (lightness), a* (green to red), b* (blue to yellow), a/b ratio, Saturation, and ∆E** (total color difference). The control bread (100% hard wheat flour) exhibited specific values for these parameters, which notably altered with the addition of FE and FT. However, our results indicate that incorporating FE and FT into Baladi bread significantly affects its color characteristics. The supplementation leads to a decrease in lightness (L* value) and an increase in redness (a*) and yellowness (b*). Specifically, bread with 30% FE supplementation showed the highest a* value, indicating increased redness, whereas the highest b* value was observed in bread with 10% FE, indicating increased yellowness. The Saturation and ∆E** values also varied, reflecting changes in color intensity and overall color difference from the control bread. Those results align with studies like those of Hussein et al.^18^ and Mezaize et al.^26^, which reported color changes in bread due to different ingredients. Similar to Aider et al.^27^ and Hadidy and Rizk^17^, additions like whey protein and coriander seeds altered the bread's color. thus, incorporating FE and FT into Baladi bread significantly influences its visual attributes, potentially affecting its sensory appeal and marketability in agreeing with Elkatry et al.^31^, Milovanović et al.^32^, and Bakare et al.^33^.

**Table 6:** Color attributes for Baladi bread supplemented with different levels of FE and FT.

Baladi bread	Color parameters					
	L*	a*	b*	a/b	Saturation	∆E**
Control (100%HWF)	64.97^a^ ± 1.01	4.86^g^ ± 0.06	17.50^c^ ± 0.32	0.28^f^ ± 0.001	18.16^e^ ± 0.19	67.46^a^ ± 0.62
90% HWF + 10% FE	52.11^c^ ± 1.05	8.65^d^ ± 0.16	18.57^a^ ± 0.14	0.47^d^ ± 0.02	20.49^b^ ± 0.13	55.99^b^ ± 0.45
80% HWF + 20%EF	39.92^e^ ± 0.56	10.97^b^ ± 0.22	15.70^f^ ± 0.07	0.70^a^ ± 0.02	19.15^d^ ± 0.15	44.28^e^ ± 0.33
70% HWF + 30% FE	33.18^g^ ± 0.85	11.20^a^ ± 0.42	17.73^b^ ± 0.18	0.63^b^ ± 0.01	20.97^a^ ± 0.07	39.25^g^ ± 0.42
90% HWF + 10% FT	53.25^b^ ± 1.45	5.99^f^ ± 0.13	15.70^f^ ± 0.22	0.38^e^ ± 0.003	16.80^g^ ± 0.16	55.84^c^ ± 0.38
80% HWF + 20% FT	42.82^d^ ± 1.20	7.44^e^ ± 0.15	16.37^e^ ± 0.26	0.45^d^ ± 0.002	17.98^f^ ± 1.15	46.44^d^ ± 0.37
70% HWF + 30% FT	38.82^f^ ± 0.65	9.60^c^ ± 0.09	17.20^d^ ± 0.12	0.56^c^ ± 0.002	19.70^c^ ± 0.08	43.53^f^ ± 0.42
LSD at 0.05	0.041	0.0430	0.145	0.0322	0.0694	0.0774

Where: HWF: Hard wheat flour; FE: *Fagopyrum esculentum*; FT:* Fagopyrum tataricum.*

Results are presented as means for triplicate analyses ± standard deviation (SD). Means within column with different letters are significantly different (*P* ≤ 0.05).

### Sensory properties of Baladi bread

The sensory properties of Baladi bread supplemented with Fagopyrum esculentum (FE) and Fagopyrum tataricum (FT) were assessed in Table 7, which is particularly significant. It provides a comprehensive view of how different levels of these supplements affect bread characteristics like appearance, layer separation, roundness, crumb distribution, crust color, taste, and odor. The results indicate that up to 20% FE or FT supplementation maintains comparable sensory qualities to the control (100% HWF), while a 30% supplementation leads to some decline in attributes like general appearance and crumb distribution. However, these findings are in line with Elkatry et al.^31^ and Milovanović et al.^32^ who found that certain alternative flours like grape seed and quinoa could improve sensory properties. Similarly, Bakare et al.^33^ observed that breadfruit flour had positive effects on sensory qualities. However, at higher substitution levels, there's a noticeable shift in sensory characteristics, which is also seen in this study at the 30% supplementation level. This aligns with Hussein et al.^18^ and Yousef et al.^20^, who noted that while barley and red algae improved nutritional quality, they also led to minor sensory changes. These results suggest a delicate balance between enhancing nutritional value and maintaining sensory acceptability, particularly at higher levels of supplementation of FE or FT.

**Table 7:** Effect of FE and FT supplementation with HWF on sensory properties of Baladi breads.

Baladi breads	General appearance (20)	Separation of layers (20)	Roundness (15)	Distribution of crumb (15)	Crust colour (10)	Taste (10)	Odour (10)
Control (100%HWF)	18.5^a^ ± 0.88	19.5^a^ ± 0.87	14.5^a^ ± 0.48	14.5^a^ ± 0.70	9.3^a^ ± 0.88	9.18^a^ ± 0.67	8.88^a^ ± 0.79
90% HWF + 10% FE	18.3^a^ ± 0.82	19.4^a^ ± 0.76	14.3^a^ ± 0.53	13.8^ab^ ± 0.67	8.3^bc^ ± 0.87	8.48^bc^ ± 0.45	8.78^ab^ ± 0.33
80% HWF + 20%FE	17.5 b ± 0.74	19.3^a^ ± 0.82	14.1^a^ ± 0.53	13.1^bc^ ± 0.82	7.5^ cd^ ± 0.82	8.38^c^ ± 0.65	8.6^bc^ ± 0.48
70% HWF + 30% FE	17.0 ^bc^ ± 0.95	19.6^a^ ± 0.65	14.2^a^ ± 0.52	12.8^c^ ± 1.17	7.5^ cd^ ± 0.95	7.58f. ± 0.49	8.4^c^ ± 0.66
90% HWF + 10% FT	18.7^a^ ± 0.92	19.2^a^ ± 0.57	14.3^a^ ± 0.42	13.4^bc^ ± 0.95	9.3^a^ ± 0.74	8.9^ab^ ± 0.52	8.18^c^ ± 0.41
80% HWF + 20% FT	18.5^a^ ± 0.64	19.1^a^ ± 0.66	14.5^a^ ± 0.48	13.5^bc^ ± 1.34	9.2^a^ ± 0.97	8.5^ cd^ ± 0.62	8.29^c^ ± 0.47
70% HWF + 30% FT	18.0 ^ab^ ± 1.08	19.3^a^ ± 0.77	14.4^a^ ± 0.88	13.3^bc^ ± 1.03	8.3^bc^ ± 0.74	8.08^ cd^ ± 0.74	8.0^b^ ± 1.15
LSD at 0.05	1.29	NS	NS	0.91	0.81	0.44	0.47

Where: HWF: Hard wheat flour; FE: *Fagopyrum esculentum*; FT:* Fagopyrum tataricum.*

Results are presented as means for triplicate analyses ± standard deviation (SD). Means within a column with different letters are significantly different (*P* ≤ 0.05)*.*

### Freshness of Baladi bread

**Figure 4:**
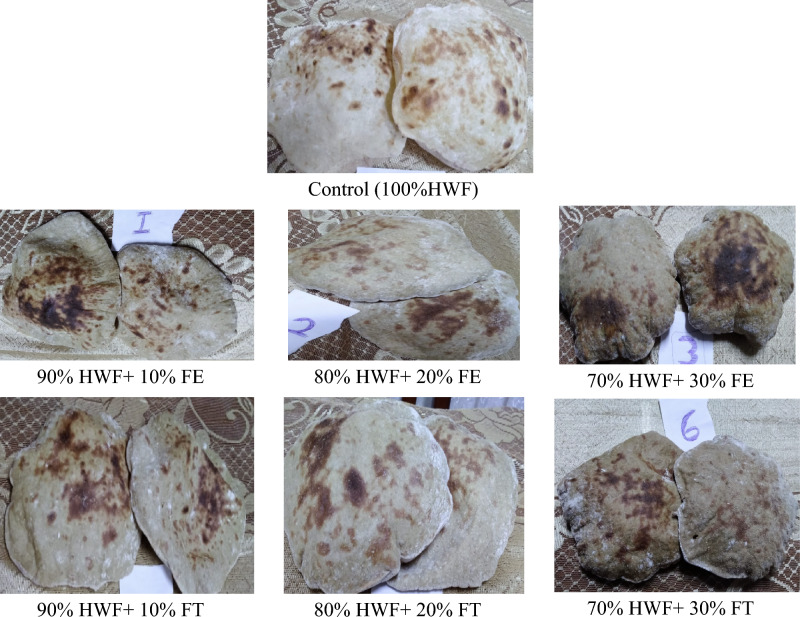
Photo of bread from hard wheat flour (HWF) supplementation with *Fagopyrum esculentum* (FE) and *Fagopyrum tataricum* (FT) at different levels.

The effect of storage period (1–3 day) at room temperature on freshness of baladi bread was evaluated as indicated in Table 8 and Fig. 4 indicate that Baladi bread supplemented with FE, particularly at 30%, shows a slower rate of staling compared to control and FT supplemented bread over 72 h. This aligns with studies on bread freshness and staling e.g., Ghoshal et al.^34^ found that enzyme supplementation, like xylanase, improves bread freshness and shelf life. El-Sayed^35^ highlighted that storage temperature influences bread freshness more than moisture content, pointing to the potential benefits of storing FT-supplemented bread at optimal temperatures. However, Wahab and Elsalam^29^ suggested higher protein content, as found in FT-supplemented bread, can improve bread freshness. Collectively, these findings indicate that supplementing Baladi bread with FT, especially at 30%, not only slows down staling but could further benefit from controlled storage conditions and enzyme or protein fortifications to maximize freshness and extend shelf life.

**Table 8:** Staling of Baladi bread supplemented with different levels of FE and FT.

Baladi bread from	Zero Time	After 24 h	After 48 h	After 72 h
Control (100%HWF)	365^e^ ± 3.60	350^d^ ± 3.70	290^bc^ ± 3.50	245^d^ ± 4.20
90% HWF + 10% FE	370^d^ ± 4.15	345^e^ ± 4.20	280^e^ ± 4.70	240^c^ ± 3.50
80% HWF + 20%FE	380^c^ ± 2.16	340^f^ ± 5.00	285^de^ ± 5.20	250^e^ ± 4.30
70% HWF + 30% FE	390^b^ ± 2.70	350^d^ ± 1.50	290^ cd^ ± 3.20	260^b^ ± 2.90
90% HWF + 10% FT	370^d^ ± 3.65	355^c^ ± 2.70	290^ cd^ ± 5.50	245^de^ ± 4.50
80% HWF + 20% FT	380^c^ ± 2.80	365^b^ ± 4.50	300^b^ ± 5.20	265^b^ ± 3.50
70% HWF + 30% FT	400^b^ ± 3.50	370^a^ ± 1.50	320^a^ ± 4.60	280^a^ ± 4.20
LSD at 0.05	1.751	1.563	5.629	3.645

Where: HWF: Hard wheat flour; FE: *Fagopyrum esculentum*; FT:* Fagopyrum tataricum.*

Results are presented as means for triplicate analyses ± standard deviation (SD). Means within column with different letters are significantly different (*P* ≤ 0.05)*.*

## Conclusion

This study confirms that supplementing Baladi bread with FE or FT leads to significant changes in nutritional profile, dough mechanics, color attributes, sensory qualities, and shelf life. Specifically, both Fagopyrum species offer nutritional benefits, such as increased protein and fiber, while imparting varied effects on dough handling and bread quality. FT, especially at 30% concentration, shows promise in slowing down the staling process, thereby potentially extending shelf life. These findings collectively highlight the utility of these alternative flours in enhancing both the nutritional and functional aspects of Baladi bread.

## Data Availability

All data generated or analysed during this study are included in this published article.
